# The predictive value of neutrophil-to-lymphocyte ratio for overall survival and pathological complete response in breast cancer patients receiving neoadjuvant chemotherapy

**DOI:** 10.3389/fonc.2022.1065606

**Published:** 2023-01-16

**Authors:** Siming Gao, Wenjie Tang, Bingli Zuo, Lianne Mulvihill, Jinming Yu, Yishan Yu

**Affiliations:** ^1^ Department of Radiation Oncology, Shandong Cancer Hospital and Institute, Shandong First Medical University and Shandong Academy of Medical Sciences, Jinan, Shandong, China; ^2^ Department of Oncology, The First Hospital of Hebei Medical University, Shijiazhuang, Hebei, China; ^3^ Department of Clinical Epidemiology and Biostatistics, Shandong Cancer Hospital and Institute, Shandong First Medical University and Shandong Academy of Medical Sciences, Jinan, Shandong, China; ^4^ Department of Radiation Oncology, Seidman Cancer Center, Case Western Reserve University School of Medicine, University Hospitals Cleveland Medical Center, Cleveland, OH, United States

**Keywords:** neutrophil-to-lymphocyte ratio, neoadjuvant chemotherapy, overall survival, pathologic complete response, nomogram, breast cancer

## Abstract

**Purpose:**

Previous studies have reported that neutrophil-to-lymphocyte ratio (NLR) at pre-treatment was predictive for overall survival (OS) and pathologic complete response (pCR) in breast cancer (BC) patients receiving neoadjuvant chemotherapy (NAC). This study aims to explore the predictive role of both pre- and post-NLR for OS as well as longitudinal NLR kinetics towards pCR in BC patients undergoing NAC.

**Methods:**

We retrospectively included 501 BC patients who received NAC from 2009 to 2018. NLR at pre-, mid (every two cycles of NAC)-, and post-treatment were collected. Overall, 421 patients were included in the survival analysis. These patients were randomly divided into a training cohort (*n* = 224) and a validation cohort (*n* = 197). A multivariable Cox model was built using all significant factors in the multivariable analysis from the training cohort. The performance of the model was verified in the validation cohort by the concordance index (C-index). Longitudinal analysis for pCR prediction of NLR was performed using a mixed-effects regression model among 176 patients who finished eight cycles of NAC.

**Results:**

The median follow-up time was 43.2 months for 421 patients. In the training cohort, multivariable analysis revealed that ER status, clinical node stage, pCR, pre-NLR, and post-NLR (all *p* < 0.05) were independent predictors of OS. The OS nomogram was established based on these parameters. The C-indexes of the nomogram were 0.764 and 0.605 in the training and validation cohorts, respectively. In the longitudinal analysis, patients who failed to achieve pCR experienced an augment of NLR during NAC while NLR remained stable among patients with pCR. Pre-NLR tended to be significantly associated with OS in patients of HER2 overexpressing and TNBC subtypes (all *p* < 0.05), but not in Luminal A and Luminal B subtypes.

**Conclusions:**

This study demonstrated the prognostic value of both pre-NLR and post-NLR on clinical outcomes in BC patients receiving NAC. A novel nomogram was established to predict OS. Non-pCR patients developed increased NLRs during NAC. Routine assessment of NLR may be a simple and affordable tool to predict prognosis for BC patients receiving NAC.

## Introduction

Breast cancer (BC) is the most common cancer and the leading cause of cancer-related death among women. It surpassed lung cancer as the leading cause of global cancer incidence in 2020. There are about 2.3 million newly diagnosed female BC cases each year, accounting for almost one in four cancer cases and one in six cancer deaths among women (1). Currently, neoadjuvant chemotherapy (NAC) serves as the standard therapy for locally advanced BC and is widely applied in the clinical setting. It works to downsize large tumors, causing the downstaging of cancer and increasing the rate of breast-conserving surgery if the BC cell is sensitive to NAC. At the same time, it can provide insight into a patient’s individual sensitivity to cancer therapies (2). Previous studies have demonstrated that patients with pathologic complete response (pCR) after NAC achieved longer overall survival (OS) compared to those without pCR after NAC (3, 4).

Recently, emerging evidence shows that the host’s systemic inflammatory response plays an important role in cancer development and progression (5–7). Some studies have found that change in the body’s inflammatory system can be indirectly reflected by the level of several immune-associated hematological indicators such as the neutrophil-to-lymphocyte ratio (NLR) (7). NLR is the ratio of the absolute neutrophil to lymphocyte count from whole blood. Neutrophils are renowned for their role in tumor proliferation and metastasis by releasing inflammatory mediators such as vascular endothelial growth factor, matrix metalloproteinase-9, and interleukin-8 (8–10). Lymphocytes are effective immune surveillance indicators, playing a key role in the cellular immunity of the body. Lymphocytes can secrete cytokines to regulate tumor immunity, which aid in immune memory and the direct killing of tumor cells. They can also suppress the proliferation and migration of tumor cells (11, 12). Therefore, elevated NLR indicates relatively high neutrophil counts and low lymphocyte counts, which may serve as an indirect reflection of impaired immunity or the burst of systematic inflammation.

NLR had been identified as an easily available biomarker in kinds of medical conditions. For example, it was reported to be an independent risk factor for COVID-19 patients (13) and determine the clinical efficacy of corticosteroid therapy among them (14). It could predict bacteremia in emergency care condition, which was better than conventional infection markers (15). NLR could also be an outcome prediction of acute intracerebral hemorrhage (16) and predict early neurological deterioration after endovascular treatment in patients with ischemic stroke (17). Some studies have also found that NLR was associated with OS among various types of cancers including colorectal cancer, lung cancer, and esophageal squamous cell carcinoma (18–21). NLR was also reported to highly predict an individual’s chemosensitivity among bladder and urothelial cancer patients (22, 23). Furthermore, several studies have found that elevated pre-treatment NLR is associated with poor survival in early triple-negative BC patients (24) and progesterone receptor (PR)/estrogen receptor (ER) positive + human epidermal growth factor receptor 2 (HER2) negative BC patients (25), predicting poor response to NAC (26).

Previous studies primarily focused on the prognostic value of pre-treatment static NLR without measuring its change during NAC (27–30). In addition, no practical survival model containing NLR has been established for BC patients in these studies. It is important to note that the tumor immune environment changes constantly during treatment. Therefore, a dynamic evaluation of NLR may be more accurate in predicting patients’ response rates toward NAC and OS. This study aims to evaluate the prognostic significance of NLR at both pre- and post-treatment as well as the role of longitudinal NLR kinetics on predicting early response to NAC in BC patients.

## Methods

### Study population

**Figure 1:**
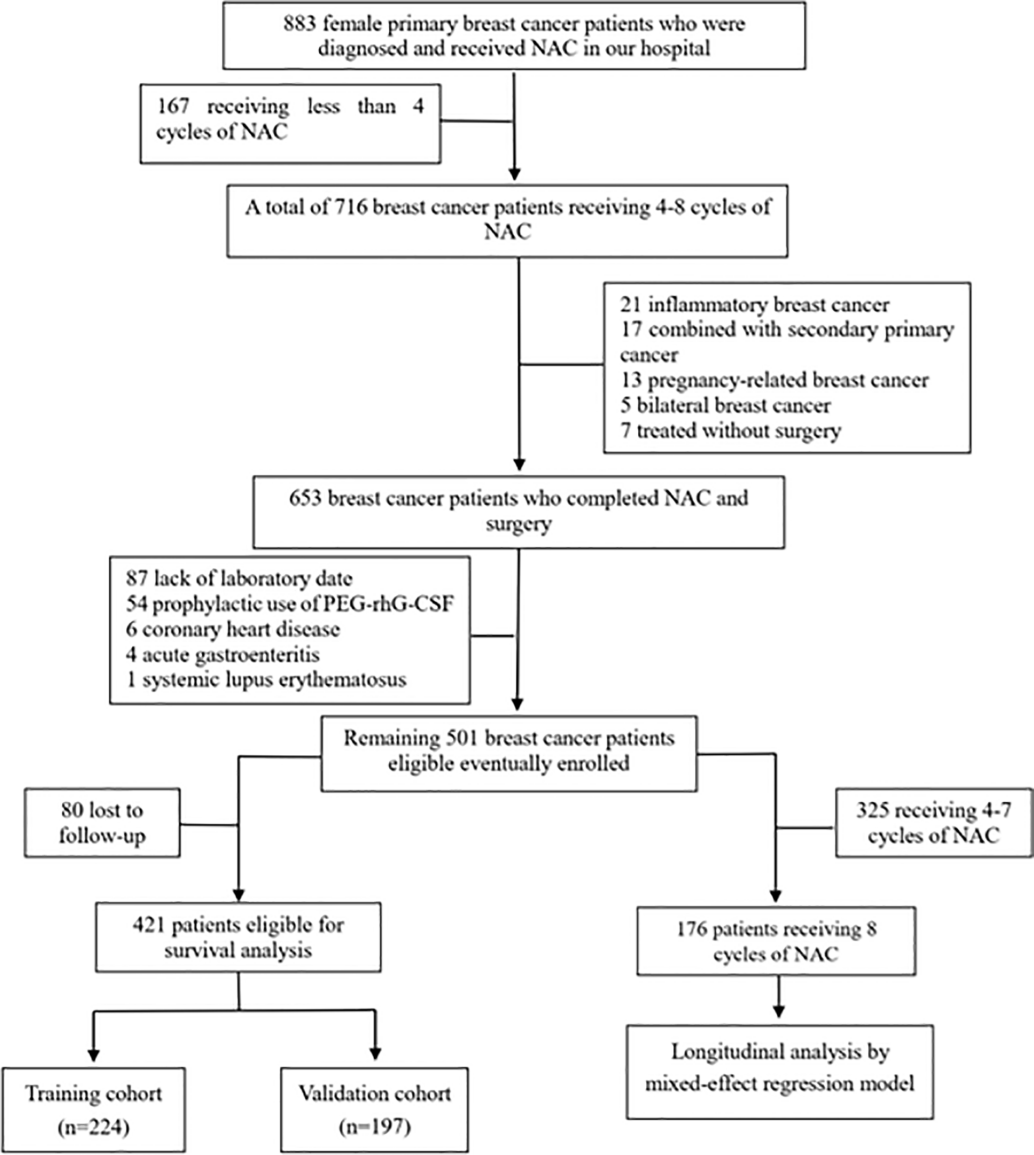
Flow diagram of the study population. NAC, neoadjuvant chemotherapy, PEG-rhG-CSF, pegylated recombinant human granulocyte colony-stimulating factor.

A total of 501 female BC patients following NAC were retrospectively identified from 2009 to 2018 using the inpatient databases from Hospital A (**Figure 1**). The following eligibility criteria were used to select the study population: (1) definitive diagnosis of invasive BC by core needle biopsy; (2) stage II–III; and (3) four to eight cycles of NAC before surgery. The exclusion criteria included the following: (1) absence of complete blood count test; (2) prophylactic use of polyethylene glycol recombinant human granulocyte colony-stimulating factor (PEG-rhG-CSF); (3) inflammatory or pregnancy-related BC; (4) bilateral BC or coexistence with other malignancies; (5) coexistence with autoimmune disease; and (6) coexistence with active infection such as acute gastroenteritis, appendicitis, or cholecystitis.

### Treatments

All patients were diagnosed pathologically. PR and ER were defined as positive when the immunohistochemistry test indicated positive invasive tumor nuclei ≥1%. HER2 positivity was defined by either a score of 3+ from immunohistochemistry or positive HER2 amplification by fluorescence *in situ* hybridization. Patients were treated according to the National Comprehensive Cancer Network (NCCN) guidelines. In this study, all patients accepted full or at least half courses of NAC. Full-course chemotherapy was defined as six to eight cycles of NAC (four cycles of anthracyclines + cyclophosphamide followed by four cycles of taxanes or six cycles of docetaxel + anthracyclines + cyclophosphamide). Half-course chemotherapy was defined as four cycles of NAC (four cycles of anthracyclines + cyclophosphamide or four cycles of taxanes + cyclophosphamide). Trastuzumab was administered to patients whose tumors were HER2-positive during a 1-year treatment period.

### Data collection

The Ethics Committee of Hospital A and Institute approved this research study. Patient information including clinicopathological data, treatment characteristics, and NLRs was collected from the electronic medical records of Hospital A. Clinicopathological factors included age, menopausal status, histologic type, hormonal status, HER2 status, Ki-67 status, clinical tumor stage, clinical lymph node status, and clinical tumor stage assessed by the American Joint Committee on Cancer (AJCC) version 7 staging system for BC. Treatment variables included the regimen of NAC, number of NAC cycles, and targeted therapy regimen. The calculated NLRs of peripheral vein blood were collected at the following time points: pre-NLR (within 3 days before the initiation of any treatment modality), mid-NLR (every two cycles during NAC), and post-NLR (completed chemotherapy and within 3 days before surgery). NLR was calculated by dividing the absolute neutrophil count by the absolute lymphocyte count.

### Statistical analysis

OS was defined as the time from the date of disease diagnosis to the date of death due to any cause or the date of the last follow-up for those alive. The “surv_cutpoint” function of the R package “Survminer” was applied for determining the optimal cutoff value of NLR, which can best dichotomize patients according to prognostic difference. Survival curves were generated by the Kaplan-Meier method and compared using the log-rank test. Patients were randomly divided into the training cohort (*n* = 224) and the validation cohort (*n* = 197) for model establishment and validation. Differences in patient characteristics and clinicopathological factors between the training and validation cohorts were determined by the Chi-squared test for categorical variables and T-test for continuous measurements. Initially, the univariate proportional hazard Cox analysis was used to assess significant predictors of OS. The variables with *p*-values < 0.150 in the univariate analysis were entered into the multivariable analysis. The variables with *p*-values < 0.050 in the multivariable analysis were incorporated into the nomogram. The above statistical evaluation was performed using the Stata/MP 15.1 (Stata Corp LP, College Station, TX). The nomogram was established and validated by “R” version 3.4.3 (http://www.r-project.org /) to predict survival rates at specific time points (3- and 5-year survivals). The performance of the nomogram model was evaluated by discrimination (C-index) and calibration curves. In addition, the nomogram model was verified again by the validation cohort.

The pCR was defined as no invasive carcinoma in the breast tissue and no residual tumor in regional lymph nodes (ypT0/ypN0). A mixed-effects regression model was used to conduct longitudinal analysis of NLR. The value of NLR was transformed to a normal distribution to better perform the regression analysis. Transformed NLR was defined as ln (NLR+0.26) using a zero-skewness log transformation in Stata/MP 15.1 (Stata Corp LP, College Station, TX). The mixed-effects regression model was performed by “R” version 3.4.3. All tests were two-sided and a *p*-value < 0.05 was considered statistically significant.

## Results

### Patient characteristics

Among the 501 BC patients enrolled in the study, 421 patients were eligible for survival analysis. The median age of these 421 patients was 49 years old and 91.2% of patients had invasive ductal carcinoma. Most patients were ER positive (61.8%), HER2 negative (65.1%), and Ki-67 ≥ 15% (76%). There were 34, 241, 63, and 79 patients at stage T1, T2, T3, and T4, respectively. Meanwhile, 58, 208, 87, and 68 patients were at clinical N0, N1, N2, and N3, respectively. Among 421 patients, 233 patients (55.3%) received ≥6 cycles of NAC. Setting OS as the state variable, the receiver operating characteristic (ROC) curve analysis was used to confirm optimal cut points for pre-NLR and post-NLR in this analysis. The cutoff values of pre-NLR and post-NLR were consequently determined as 2.2 and 2.7, respectively. Patients’ baseline characteristics in the training cohort were comparable to those in the validation cohort (**Table 1**). The median follow-up time was 43.2 months (2.9–114.7 months).

**Table 1:** Comparison of patient characteristics between training and validation cohort.

Variables	All patients	Training cohort	Validation cohort	p-value
	N = 421	N = 224	N = 197	
Age (years), n (%)				
≤49	221 (52.5)	117 (52.2)	104 (52.8)	0.909
>49	200 (47.5)	107 (47.8)	93 (47.2)	
Menopause, n (%)				
Yes	180 (42.8)	97 (43.3)	83 (42.1)	0.808
No	241 (57.2)	127 (56.7)	114 (57.9)	
Histology, n (%)				
Ductal	384 (91.2)	204 (91.1)	180 (91.4)	0.914
Lobular/Others	37 (8.8)	20 (8.9)	17 (8.6)	
ER status, n (%)				
Positive	260 (61.8)	137 (61.2)	123 (62.4)	0.788
Negative	161 (38.2)	87 (38.8)	74 (37.6)	
PR status, n (%)				
Positive	227 (53.9)	116 (51.8)	111 (56.3)	0.349
Negative	194 (46.1)	108 (48.2)	86 (43.7)	
HER2 status, n (%)				
Negative	274 (65.1)	148 (64.7)	126 (64)	0.869
Positive	147 (34.9)	79 (35.3)	71 (36)	
Ki-67, n (%)				
<15%	101 (24)	51 (23)	50 (25.4)	0.565
≥15%	320 (76)	171 (77)	147 (74.6)	
Clinical tumor stage, n (%)				
cT1	34 (8.1)	15 (6.7)	19 (9.6)	0.670
cT2	241 (57.2)	131 (60.3)	110 (55.8)	
cT3	63 (14.9)	33 (14.7)	30 (15.2)	
cT4	79 (18.8)	41 (18.3)	38 (19.3)	
Clinical lymph node stage, n (%)				
cN0	58 (13.8)	27 (12.1)	31 (15.7)	0.499
cN1	208 (49.4)	108 (48.2)	100 (50.8)	
cN2	87 (20.7)	49 (21.9)	38 (19.3)	
cN3	68 (16.1)	40 (17.9)	28 (14.2)	
Clinical stage (AJCC 7th), n (%)				
Stage II	198 (47)	102 (45.5)	96 (48.7)	0.512
Stage III	223 (53)	122 (54.5)	101 (51.3)	
Cycles of chemotherapy, n (%)				
<6	198 (47)	107 (47.8)	91 (46.2)	0.747
≥6	223 (53)	117 (52.2)	106 (53.8)	
Pre-NLR				
≤2.2	242 (33.7)	127 (56.7)	115 (58.4)	0.728
>2.2	179 (66.3)	97 (43.3)	82 (41.6)	
Post-NLR				
≤2.7	222 (52.7)	118 (52.7)	104 (52.8)	0.981
>2.7	199 (47.3)	106 (47.3)	93 (47.2)	

ER, estrogen receptor; PR, progesterone receptor; HER2, human epidermal growth factor receptor; AJCC, American Joint Committee on Cancer; NLR, neutrophil-to-lymphocyte ratio.

### The predictive value of NLR in different BC subtypes

Four BC subtypes were determined according to the ER/PR/HER2 status: ER+/HER2− or PR+/HER2− (Luminal A), ER+/HER2+ or PR+/HER2+ (Luminal B), ER−/PR−/HER2+ (HER2 overexpressing), and ER−/PR−/HER2− [triple-negative breast cancer (TNBC)]. The distribution of the Luminal A, Luminal B, HER2 overexpressing, and TNBC subtypes were 47.5%, 18.8%, 16.9%, and 16.9%, respectively. By univariate and multivariable proportional hazard Cox analysis, the variable of pre-NLR tended to be significantly associated with OS in patients of HER2 overexpressing and TNBC subtypes (all *p* < 0.05), but not in Luminal A and Luminal B subtypes. Post-NLR only made sense in patients with HER2 overexpressing (*p* = 0.016).

### Univariate analysis in the training cohort

**Figure 2:**
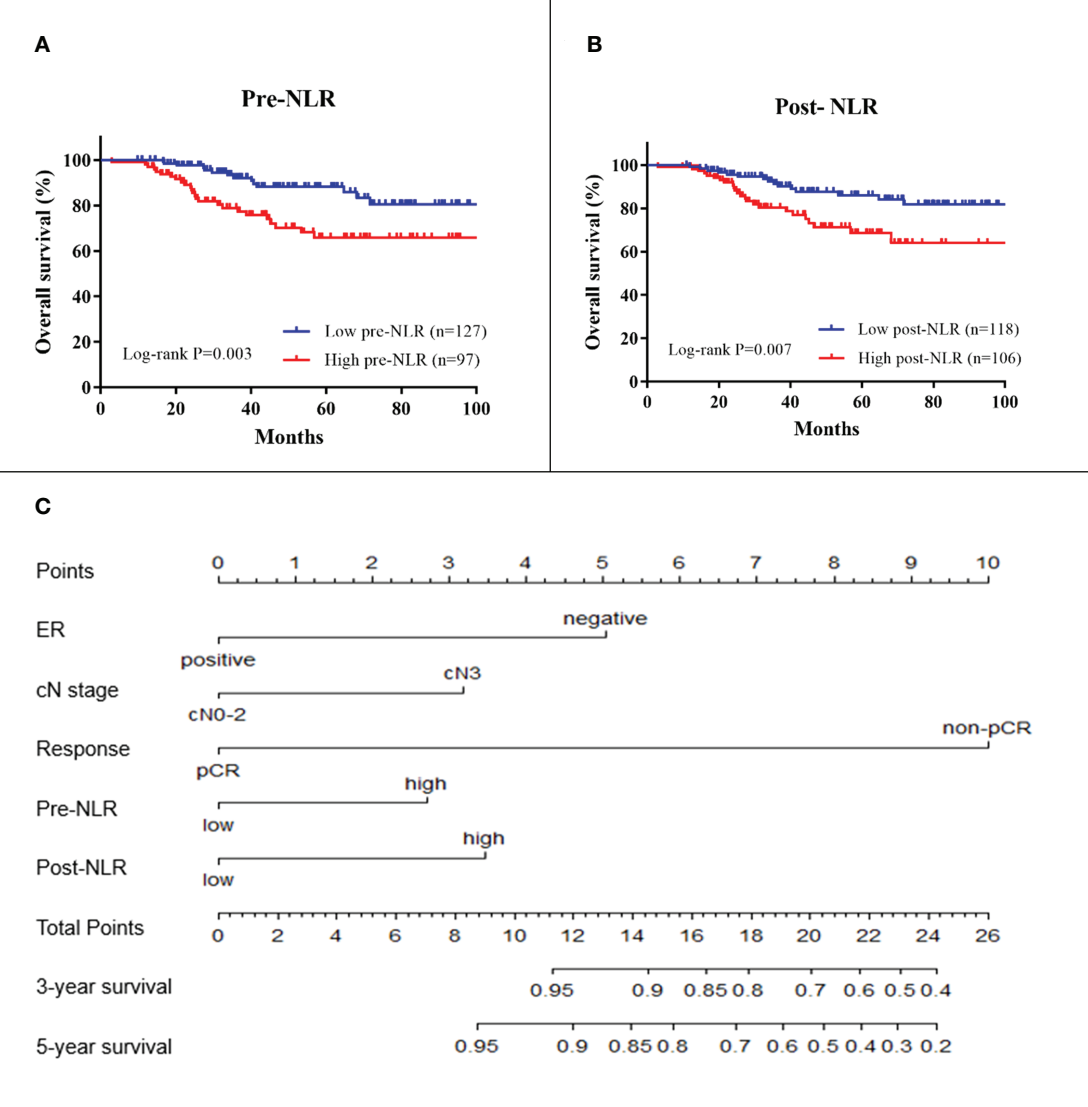
Kaplan–Meier survival plots of pre-NLR **(A)** and post-NLR **(B)** for overall survival. A nomogram **(C)** for predicting survival in breast cancer patients receiving NAC. ER, estrogen receptor; cN, clinical lymph node; NLR, neutrophil-to-lymphocyte ratio. Response is the response to neoadjuvant chemotherapy.

In the training cohort, the results of univariate analysis showed that ER status, HER2 status, clinical node stage, pCR, pre-NLR, and post-NLR were significantly associated with OS (all *p* < 0.05) (**Table 2**). The 5-year OS in the low and high pre-NLR patients were 88.2% and 65.9%, respectively (*p* = 0.003, **Figure 2**). The 5-year OS in the low and high post-NLR patients were 86.0% and 68.6%, respectively (*p* = 0.007, **Figure 2**).

**Table 2:** Univariate and multivariable analyses of OS in the training cohort using the Cox model.

Variables	Univariate analysis			Multivariable analysis		
	HR	95% CI	*p*-value	HR	95% CI	*p*-value
Age (years)						
≤49	1.000 (ref.)					
>49	1.580	0.873–2.981	0.158			
Menopause						
Yes	1.000 (ref.)					
No	1.151	0.603–2.198	0.670			
Histology						
Ductal	1.000 (ref.)					
Lobular/Others	0.727	0.175–3.027	0.662			
ER status						
Positive	1.000 (ref.)			1.000 (ref.)		
Negative	2.461	1.278–4.737	**0.007**	3.480	1.333–9.080	**0.011**
PR status						
Positive	1.000 (ref.)			1.000 (ref.)		
Negative	1.696	0.889–3.235	**0.109**	0.672	0.262–1.719	0.407
HER2 status						
Negative	1.000 (ref.)			1.000 (ref.)		
Positive	2.056	1.091–3.873	**0.026**	1.568	0.796–3.089	0.193
Ki-67						
<15%	1.000 (ref.)					
≥15%	1.621	0.711–3.699	0.251			
Clinical tumor stage						
cT1	1.000 (ref.)					
cT2–4	1.301	0.313–5.402	0.717			
Clinical node stage						
cN0–2	1.000 (ref.)			1.000 (ref.)		
cN3	2.540	1.252–5.152	**0.010**	2.108	1.020–4.356	**0.044**
Cycles of chemotherapy						
<6	1.000 (ref.)					
≥6	1.066	0.563–2.019	0.844			
pCR						
No	1.000 (ref.)			1.000 (ref.)		
Yes	0.134	0.018–0.977	**0.047**	0.119	0.016–0.887	**0.038**
Pre-NLR						
≤2.2	1.000 (ref.)			1.000 (ref.)		
>2.2	2.609	1.356–5.020	**0.004**	1.962	1.002–3.842	**0.049**
Post-NLR						
≤2.7	1.000 (ref.)			1.000 (ref.)		
>2.7	2.385	1.246–4.567	**0.009**	2.133	1.088–4.182	**0.027**

95% CI, 95% confidence interval; HR, hazard ratio; ER, estrogen receptor; PR, progesterone receptor; HER2, human epidermal growth factor receptor; pCR, pathologic complete response; NLR, neutrophil-to-lymphocyte ratio. Meaningful values are highlighted in bold.

### Multivariable analysis and nomogram development

**Figure 3:**
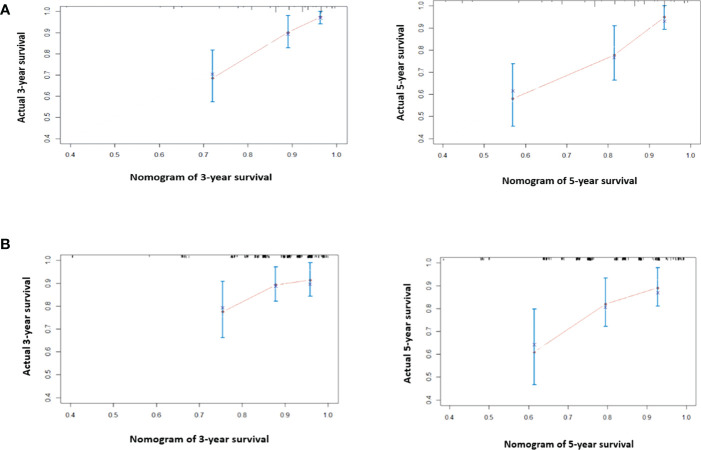
The calibration curve for predicting 3- and 5-year survival in the training cohort **(A)** and validation cohort **(B)**. Nomogram-predicted probability of overall survival is plotted on the *x*-axis; true overall survival is plotted on the *y*-axis. The predicted probability is almost the same as the true probability. The prediction error of the model is acceptable.

For model building, clinical variables with *p*-values < 0.150 in the univariate analysis including ER status, PR status, HER2 status, clinical node stage, pCR, pre-NLR, and post-NLR were selected as candidate variables for the multivariable Cox model. Finally, five variables including ER status, clinical node stage, pCR, pre-NLR, and post-NLR (all *p* < 0.05) were selected into the survival model in the multivariable analysis **Table 2**). The nomogram predicting 3- and 5-year survival is shown in **Figure 2**. The C-indexes of the nomogram in the training and validation cohorts were 0.764 (95% CI 0.692–0.836) and 0.605 (95% CI 0.541–0.669), respectively. The calibration plots of the nomogram showed good agreement between the prediction by nomogram and actual observation in both the training (**Figure 3**) and validation (**Figure 3**) cohorts for 3- and 5-year OS.

### Mixed-effects regression analysis of NLR during NAC

**Figure 4:**
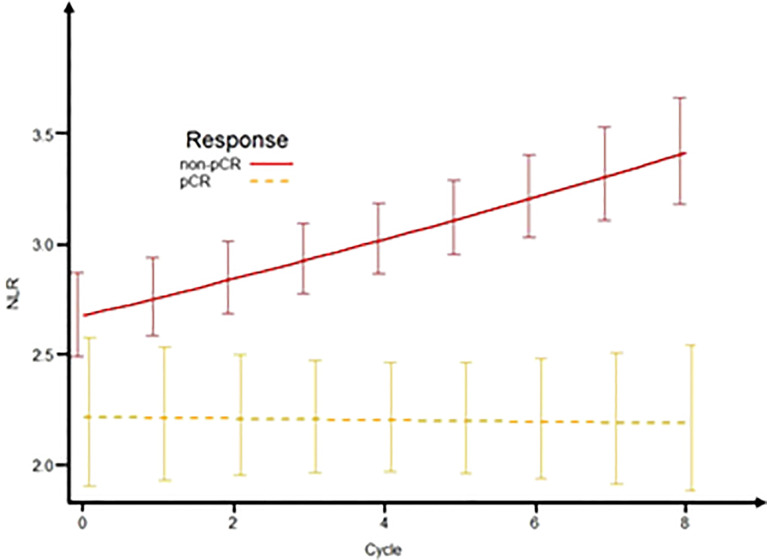
Predictive NLR over time according to response status. The plot of the mixed-effects model to predict NLR by response status (pCR and non-pCR) over time. NLR, neutrophil-to-lymphocyte ratio; pCR, pathologic complete response.

Of the 501 patients enrolled, 176 patients received eight cycles of NAC. These patients were eligible for longitudinal analysis of NLR by mixed-effects regression analysis. We applied the mixed-effects regression model to predict NLR by response category (**Figure 4**). The results showed that patients with non-pCR on NAC had an increasing NLR trend over time while NLR remained unchanged in the group of pCR patients. Compared with the pCR group, non-pCR patients had an average of +0.032 change in transformed NLR every two cycles of NAC (std error = 0.014, *p* = 0.024).

## Discussion

In this retrospective study including a total of 501 patients who received NAC, we observed that (1) both the pre- and post-NLR were significantly associated with OS; (2) besides the NLRs, three clinical characteristics (ER status, clinical node stage, and pCR) were also identified as independent prognostic factors for BC patients; (3) a predictive nomogram model for OS including all significant factors showed reasonable discriminations and calibrations; (4) longitudinal analysis of NLR revealed an increasing NLR trend over time for patients with non-pCR on NAC compared to unchanged NLR for patients with pCR; and (5) the predictive value of NLR varies in different BC subtypes. pre-NLR could predict the survival of patients in HER2 overexpressing and TNBC subtypes, but not in Luminal A and Luminal B subtypes. Post-NLR only made sense in patients with HER2 overexpressing. Overall, this study demonstrated the prognostic role of NLRs and its strong relationship with pCR for BC patients receiving NAC.

Recently, elevated NLR was reported to be associated with poor survival in patients diagnosed with several types of cancer (18–21). The prognostic value of pre-NLR on survival had been evaluated in several studies, which showed inconsistent results in BC patients (29, 31–34). Some studies found that the prognostic value of pre-NLR depended on the phenotype of BC. A large-scale study involving 1,519 BC patients indicated that patients with higher NLR had worse OS in the presence of triple-negative BC as well as HER2-overexpressing BC (33). Other studies also found an association between NLR and survival in patients with HER2-positive BC (34) and HER2−negative BC (35). However, not all studies had similar conclusions. Alejandra et al. found that pre-NLR was not an independent prognostic predictor for metastatic breast cancer (MBC) (36). This inconsistency might be due to the late clinical stage of BC and the small sample size of the study. The results of our study, which included a large sample size, support the prognostic value of pre-NLR in locally advanced BC patients receiving NAC. Similar to previous studies, we also found that the prognostic value of pre-NLR might depend on the subtypes of BC. The pre-NLR might predict the survival of patients in HER2 overexpressing and TNBC subtypes better. Moreover, our results demonstrated that high NLR after NAC treatment could also be an independent predictor for OS. The prognostic value of post-NLR had been identified by previous investigations in other carcinomas such as non-small cell lung cancer (37) and head–neck cancer (38). Compared with pre-NLR, we believe that post-NLR may indicate the changed tumor immune environment after NAC. As mentioned above, elevated NLR indicates relatively low lymphocyte counts and high neutrophil counts. Previous studies had also demonstrated that post-treatment lymphocyte depletion and opportunistic infections are associated with reduced survival in cancer patients (39), which may explain the mechanism of why high post-NLR in BC patients is associated with poor prognosis. More prospective clinical studies are needed to further illustrate the prognostic value of post-NLR and validate this relation.

Besides pre-NLR and post-NLR, three clinicopathological characteristics (clinical N0–2 stage, pCR, and ER-positive) predicted improved OS, which were consistent with previous studies (3, 40, 41). Clinical N0–2 stage indicated earlier stage, which is definitively associated with better survival outcomes (40). Patients with pCR typically do not have any residual invasive cancers, which also represents a better therapeutic result of NAC (41). ER-positive BC is a more favorable phenotype because patients usually have the opportunity to be treated with endocrine therapy. In our study, a novel nomogram model was established to predict the OS of BC patients treated with NAC based on the pre- and post-NLRs along with these three clinicopathological characteristics. Nomogram models are widely utilized in cancer prognosis due to their ability to transform statistical results into a visual quantitative assessment of the risk of an event, typically death or recurrence. Currently, there are only a few nomogram models for DFS in BC patients receiving NAC (42, 43). Our nomogram model involved a relatively larger sample size compared to those of previous studies. This nomogram is the first OS model construction to incorporate NLR along with clinicopathological factors for BC patients treated with NAC. Both the training and validation cohorts showed great discrimination power (C-index, 0.764 and 0.605) when NLR was included. Nomogram as a simple graphical prediction model can be a good way to show the influence of each significant variable in one prediction model. We believe that this model can be applied as an assessment tool and therapeutic guidance for BC using the easily accessible parameters once validated by more patient cohorts in future studies.

In our study, there were 176 patients who completed eight cycles of NAC that were available for longitudinal analysis of NLR by mixed-effects regression analysis. The whole blood count information of this cohort at five time points (pre-treatment and treatment after the 2nd, 4th, 6th, and 8th cycles of NAC) were analyzed. The results showed that delta-NLR was significantly associated with the individual’s response to chemotherapy. We found that NLR in patients with pCR were stable, while patients with non-pCR showed an increase of NLR during NAC. Accordingly, dynamic monitoring of NLR during NAC among BC patients may be helpful for identifying patients who can benefit from NAC. A significant increase of NLR is a signal of the impaired immune system during the process of treatment, which may be used as an early indicator of the NAC resistance before image detection in the clinic. Due to the complex nature of carcinoma, the progression according to time has always been changing. Therefore, the persistent estimation of NLR can provide additive values for early evaluation of NAC. A routine blood test is widely used as a traditional examination test in most cancer patients, especially in the process of NAC. Several studies have reported the prognostic value of delta-NLR and pCR among BC patients (44, 45). Based on these results, we propose that NLR might be an easy and inexpensive tool predicting the response to NAC in BC patients. However, these findings still need to be validated by large prospective trials in the future.

Despite the novelty and potential of our study, there are some limitations that must be addressed. Firstly, this is a single-center study. Although we have a large cohort of 501 patients, an additional external validation would make the results more generalizable. NLR may be influenced by many other factors. As a result, we excluded the influence of infection and steroid use in our study to minimize confounding factors. The retrospective nature of this study should warrant future prospective studies to validate these results. Nevertheless, we believe that the predictive trend of NLR will not change significantly even in other prospective studies.

## Conclusions

This study revealed the prognostic value of pre-NLR and post-NLR for OS in BC patients treated with NAC. A novel nomogram containing pre-NLR, post-NLR, clinical N stage, ER status, and pCR was established to predict the 3- and 5-year OS of BC patients, which can be used in patient consultations, assessment of prognosis, and further treatment strategies. The predictive value of NLR varies in different BC subtypes, which should be noted in clinical application. Longitudinal analysis of NLR revealed an increasing NLR trend for non-pCR patients compared to unchanged NLR for pCR patients during NAC. Routine assessment of this parameter may be an easy and affordable tool for predicting prognosis of BC.

## Data availability statement

The raw data supporting the conclusions of this article will be made available by the authors, without undue reservation. The software application or custom code will be made available by the corresponding author upon reasonable request.

## Ethics statement

This study was approved by the Ethics Committee of Shandong Cancer Hospital and Institute. Consent to participate was obtained from all included individuals. Written informed consent was obtained from the individual(s) for the publication of any potentially identifiable images or data included in this article.

## Author contributions

All authors contributed to the study conception and design. Material preparation, data collection, and analysis were performed by SG and WT. The first draft of the manuscript was written by SG and WT. Writing—review and editing was performed by LM and YY. Project administration and funding acquisition were carried out by JY. All authors contributed to the article and approved the submitted version.

## References

[1] Sung H Ferlay J Siegel RL Laversanne M Soerjomataram I Jemal A. Global cancer statistics 2020: GLOBOCAN estimates of incidence and mortality worldwide for 36 cancers in 185 countries. CA Cancer J Clin (2021) 71(3):209–49. doi: 10.3322/caac.21660 33538338

[2] Fisher B Brown A Mamounas E Wieand S Robidoux A Margolese RG. Effect of preoperative chemotherapy on local-regional disease in women with operable breast cancer: findings from national surgical adjuvant breast and bowel project b-18. J Clin Oncol (1997) 16(7):2483–93. doi: 10.1200/JCO.1997.15.7.2483 9215816

[3] Cortazar P Zhang L Untch M Mehta K Costantino JP Wolmark N. Pathological complete response and long-term clinical benefit in breast cancer: the CTNeoBC pooled analysis. Lancet (2014) 384(9938):164–72. doi: 10.1016/S0140-6736(13)62422-8 24529560

[4] von Minckwitz G Untch M Blohmer JU Costa SD Eidtmann H Fasching PA. Definition and impact of pathologic complete response on prognosis after neoadjuvant chemotherapy in various intrinsic breast cancer subtypes. J Clin Oncol (2012) 30(15):1796–804. doi: 10.1200/JCO.2011.38.8595 22508812

[5] Fernandes JV Cobucci RN Jatoba CA Fernandes TA de Azevedo JW de Araujo JM. The role of the mediators of inflammation in cancer development. Pathol Oncol Res (2015) 21(3):527–34. doi: 10.1007/s12253-015-9913-z 25740073

[6] Bremnes RM Al-Shibli K Donnem T Sirera R Al-Saad S Andersen S. The role of tumor-infiltrating immune cells and chronic inflammation at the tumor site on cancer development, progression, and prognosis: Emphasis on non-small cell lung cancer. J Thorac Oncol (2010) 6(4):824–33. doi: 10.1097/JTO.0b013e3182037b76 21173711

[7] Hanahan D Weinberg RA. Hallmarks of cancer: the next generation. Cell (2011) 144(5):646–74. doi: 10.1016/j.cell.2011.02.013 21376230

[8] Kusumanto YH Dam WA Hospers GAP Meijer C Mulder NH. Platelets and granulocytes, in particular the neutrophils, form important compartments for circulating vascular endothelial growth factor. Angiogenesis (2003) 6(4):283–7. doi: 10.1023/B:AGEN.0000029415.62384.ba 15166496

[9] Bausch D Pausch T Krauss T Hopt UT Fernandez-del-Castillo C Warshaw AL. Neutrophil granulocyte derived MMP-9 is a VEGF independent functional component of the angiogenic switch in pancreatic ductal adenocarcinoma. Angiogenesis (2011) 14(3):235–43. doi: 10.1007/s10456-011-9207-3 PMC3688040 21442180

[10] De Larco JE Wuertz BRK Furcht LT. The potential role of neutrophils in promoting the metastatic phenotype of tumors releasing interleukin-8. Clin Cancer Res an Off J Am Assoc Cancer Res (2004) 10(15):4895. doi: 10.1158/1078-0432.CCR-03-0760 15297389

[11] Mantovani A Allavena P Sica A Balkwill F. Cancer-related inflammation. Nature (2008) 454(7203):436–44. doi: 10.1038/nature07205 18650914

[12] Carbone DP Gandara DR Antonia SJ Zielinski C Paz-Ares L. Non-Small-Cell lung cancer: Role of the immune system and potential for immunotherapy. J Thorac Oncol (2015) 10(7):974–84. doi: 10.1097/JTO.0000000000000551 PMC4618296 26134219

[13] Yuwei L Xuebei D Jing C Yalei J Li P Harry HXW. Neutrophil-to-lymphocyte ratio as an independent risk factor for mortality in hospitalized patients with COVID-19. J Infect (2020) 81(1):e6–e12. doi: 10.1016/j.jinf.2020.04.002 PMC7195072 32283162

[14] Jingjing C Haomiao L Changjiang Z Ze C Hui L Fang L. The neutrophil-to-Lymphocyte ratio determines clinical efficacy of corticosteroid therapy in patients with COVID-19. Cell Metab (2021) 33(2):258–269.e3. doi: 10.1016/j.cmet.2021.01.002 33421384 PMC7832609

[15] de Jager CPC van Wijk PTL Mathoera RB de Jongh-Leuvenink J van der Poll T. Lymphocytopenia and neutrophil-lymphocyte count ratio predict bacteremia better than conventional infection markers in an emergency care unit. Comp Study Crit Care (2010) 14(5):R192. doi: 10.1186/cc9309 PMC3219299 21034463

[16] Lattanzi S Cagnetti C Rinaldi C Angelocola S Provinciali L. Neutrophil-to-lymphocyte ratio improves outcome prediction of acute intracerebral hemorrhage. J Neurol Sci (2018) 387:98–102. doi: 10.1016/j.jns.2018.01.038 29571881

[17] Lattanzi S Norata D Broggi S Meletti S Świtońska M. Neutrophil-to-Lymphocyte ratio predicts early neurological deterioration after endovascular treatment in patients with ischemic stroke. Life (Basel) (2022) 12(9):1415. doi: 10.3390/life12091415 36143451 PMC9503346

[18] Climent M Ryan EJ Stakelum A Khaw YL Creavin B Lloyd A. Systemic inflammatory. response predicts oncological outcomes in patients undergoing elective surgery for mismatch repair-deficient colorectal cancer. Int J Colorectal Dis (2019) 34(6):1069–78. doi: 10.1007/s00384-019-03274-6 30993458

[19] Luo G Guo M Liu Z Xiao Z Jin K Long J. Blood neutrophil-lymphocyte ratio predicts survival in patients with advanced pancreatic cancer treated with chemotherapy. Ann Surg Oncol (2015) 22(2):670–6. doi: 10.1245/s10434-014-4021-y 25155401

[20] Bar-Ad V Palmer J Li L Lai Y Lu B Myers RE. Neutrophil to lymphocyte ratio associated with prognosis of lung cancer. Clin Transl Oncol (2017) 19(6):711–7. doi: 10.1007/s12094-016-1593-y 27909873

[21] Chen MF Chen PT Kuan FC Chen WC. The predictive value of pretreatment neutrophil-To-Lymphocyte ratio in esophageal squamous cell carcinoma. Ann Surg Oncol (2019) 26(1):190–9. doi: 10.1245/s10434-018-6944-1 30362062

[22] Seah JA Leibowitz-Amit R Atenafu EG Alimohamed N Knox JJ Joshua AM. Neutrophil-lymphocyte ratio and pathological response to neoadjuvant chemotherapy in patients with muscle-invasive bladder cancer. Clin Genitourin Cancer (2015) 13(4):e229–33. doi: 10.1016/j.clgc.2015.02.001 25777682

[23] Rossi L Santoni M Crabb SJ Scarpi E Burattini L Chau C. High neutrophil-to-lymphocyte ratio persistent during first-line chemotherapy predicts poor clinical outcome in patients with advanced urothelial cancer. Ann Surg Oncol (2015) 22(4):1377–84. doi: 10.1245/s10434-014-4097-4 25234022

[24] Pistelli M Lisa MD Ballatore Z Caramanti M Pagliacci A Battelli N. Pre-treatment neutrophil to lymphocyte ratio may be a useful tool in predicting survival in early triple negative breast cancer patients. BMC Cancer (2015) 15(1):195. doi: 10.1186/s12885-015-1204-2 25884918 PMC4428113

[25] Koh YW Lee HJ Ahn JH Lee JW Gong G. Prognostic significance of the ratio of absolute neutrophil to lymphocyte counts for breast cancer patients with ER/PR-positivity and HER2-negativity in neoadjuvant setting. Tumour Biol (2014) 35(10):9823–30. doi: 10.1007/s13277-014-2282-5 24986572

[26] Chen Y Chen K Xiao X Nie Y Qu S Gong C. Pretreatment neutrophil-to-lymphocyte ratio is correlated with response to neoadjuvant chemotherapy as an independent prognostic indicator in breast cancer patients: a retrospective study. BMC Cancer (2016) 16:320. doi: 10.1186/s12885-016-2352-8 27198767 PMC4872336

[27] Noh H Eomm M Han A. Usefulness of pretreatment neutrophil to lymphocyte ratio in predicting disease-specific survival in breast cancer patients. J Breast Cancer (2013) 16(1):55–9. doi: 10.4048/jbc.2013.16.1.55 PMC3625770 23593082

[28] Asano Y Kashiwagi S Onoda N Noda S Kawajiri H Takashima T. Predictive value of Neutrophil/Lymphocyte ratio for efficacy of preoperative chemotherapy in triple-negative breast cancer. Ann Surg Oncol (2016) 23(4):1104–10. doi: 10.1245/s10434-015-4934-0 PMC4773470 26511266

[29] Azab B Bhatt VR Phookan J Murukutla S Kohn N Terjanian T. Usefulness of the neutrophil-to-lymphocyte ratio in predicting short- and long-term mortality in breast cancer patients. Ann Surg Oncol (2012) 19(1):217–24. doi: 10.1245/s10434-011-1814-0 21638095

[30] Junttila MR de Sauvage FJ. Influence of tumour micro-environment heterogeneity on therapeutic response. Nature (2013) 501(7467):346–54. doi: 10.1038/nature12626 24048067

[31] Azab B Shah N Radbel J Tan P Bhatt V Vonfrolio S. Pretreatment neutrophil/lymphocyte ratio is superior to platelet/lymphocyte ratio as a predictor of long-term mortality in breast cancer patients. Med Oncol (2013) 30(1):432. doi: 10.1007/s12032-012-0432-4 23283648

[32] Gago-Dominguez M Matabuena M Redondo CM Patel SP Carracedo A Ponte SM. Neutrophil to lymphocyte ratio and breast cancer risk: analysis by subtype and potential interactions. Sci Rep (2020) 10(1):13203. doi: 10.1038/s41598-020-70077-z 32764699 PMC7413522

[33] Muñoz-Montaño W Cabrera-Galeana P Alvarado-Miranda A Villareal-Garza C Mohar A Olvera A. Prognostic value of the pretreatment neutrophil-to-Lymphocyte ratio in different phenotypes of locally advanced breast cancer during neoadjuvant systemic treatment. Clin Breast Cancer (2020) 20(4):307–16. doi: 10.1016/j.clbc.2019.12.011 32305297

[34] Li Y Shao Y Bai L Zhuo X. Increased derived neutrophil-to-lymphocyte ratio and breast imaging-reporting and data system classification predict poor survival in patients with non-distant metastatic HER2+ breast cancer treated with neoadjuvant chemotherapy. Cancer Manage Res (2018) 9(10):3841–7. doi: 10.2147/CMAR.S174537 PMC6161733 30288115

[35] June Bae S Jin Cha Y Yoon C Kim D Lee J Park S. Prognostic value of neutrophil−to−lymphocyte ratio in human epidermal growth factor receptor 2−negative breast cancer patients who received neoadjuvant chemotherapy affiliations. Sci Rep (2020) 10(1):13078. doi: 10.1038/s41598-020-69965-1 32753659 PMC7403312

[36] Ivars Rubio A Yufera JC de la Morena P Fernández Sánchez A Navarro Manzano E García Garre E. Neutrophil-lymphocyte ratio in metastatic breast cancer is not an independent predictor of survival, but depends on other variables. Sci Rep (2019) 9(1):16979. doi: 10.1038/s41598-019-53606-3 31740715 PMC6861311

[37] Jin F Han A Shi F Kong L Yu J. The postoperative neutrophil-to-lymphocyte ratio and changes in this ratio predict survival after the complete resection of stage I non-small cell lung cancer. Onco Targets Ther (2016) 9:6529–37. doi: 10.2147/OTT.S117290 PMC5085302 27799800

[38] Kim DY Kim IS Park SG Kim H Choi YJ Seol YM. Prognostic value of posttreatment neutrophil-lymphocyte ratio in head and neck squamous cell carcinoma treated by chemoradiotherapy. Auris Nasus Larynx (2017) 44(2):199–204. doi: 10.1016/j.anl.2016.05.013 27269133

[39] Yovino S Kleinberg L Grossman SA Narayanan M Ford E. The etiology of treatment-related lymphopenia in patients with malignant gliomas: modeling radiation dose to circulating lymphocytes explains clinical observations and suggests methods of modifying the impact of radiation on immune cells. Cancer Invest (2013) 31(2):140–4. doi: 10.3109/07357907.2012.762780 PMC3991115 23362951

[40] Liao GS Chou YC Golshan M Hsu HM Hong ZJ Yu JC. Prognostic value of the lymph node ratio in breast cancer subtypes. Am J Surg (2015) 210(4):749–54. doi: 10.1016/j.amjsurg.2014.12.054 26130268

[41] Yang Y Wang Y Deng H Tan C Li Q He Z. Development and validation of nomograms predicting survival in Chinese patients with triple negative breast cancer. BMC Cancer (2019) 19(1):541. doi: 10.1186/s12885-019-5703-4 31170946 PMC6555047

[42] Lai J Pan Z Chen P Ye G Chen K Su F. Development and validation of a nomogram incorporating axillary lymph node ratio to predict survival in node-positive breast cancer patients after neoadjuvant chemotherapy. Jpn J Clin Oncol (2019) 49(1):22–8. doi: 10.1093/jjco/hyy181 30508184

[43] Colleoni M Bagnardi V Rotmensz N Dellapasqua S Viale G Pruneri G. A risk score to predict disease-free survival in patients not achieving a pathological complete remission after preoperative chemotherapy for breast cancer. Ann Oncol (2009) 20(7):1178–84. doi: 10.1016/j.breast.2012.06.003 19218304

[44] Dan JQ Tan JY Huang JH Zhang XL Guo Y Huang YK. The dynamic change of neutrophil to lymphocyte ratio is predictive of pathological complete response after neoadjuvant chemotherapy in breast cancer patients. Breast Cancer (2020) 27(5):982–8. doi: 10.1007/s12282-020-01096-x 32306184

[45] Kim J-Y Jung Jung E Kim JM Shin Lee H Kwag SJ Park JH. Dynamic changes of neutrophil-to-lymphocyte ratio and platelet-to-lymphocyte ratio predicts breast cancer prognosis. BMC Cancer (2020) 20(1):1206. doi: 10.1186/s12885-020-07700-9 33287745 PMC7720486

